# Inverted Lymphoglandular Polyp in Descending Colon

**DOI:** 10.1155/2015/646270

**Published:** 2015-02-12

**Authors:** Shengmei Zhou, Yanling Ma, Parakrama Chandrasoma

**Affiliations:** ^1^Department of Pathology and Laboratory Medicine, Children's Hospital Los Angeles, Los Angeles, CA 90027, USA; ^2^Keck School of Medicine, University of Southern California, Los Angeles, CA 90033, USA; ^3^Department of Pathology, LAC+USC Medical Center, Los Angeles, CA 90033, USA

## Abstract

A 47-year-old male with a history of left colon cancer, status post left colon resection for 12 years, presented with rectal bleeding. Colonoscopic examination revealed an 8 mm sessile polyp in the proximal descending colon. Microscopic examination showed that the surface of this polyp was covered with a layer of normal colonic mucosa with focal surface erosion. In the submucosal layer, an intimate admixture of multiple cystically dilated glands and prominent lymphoid aggregates with germinal centers was seen. The glands were lined by columnar epithelium. Immunohistochemical staining showed the glands were positive for CK20 and CDX2 and negative for CK7, with a low proliferative index, mostly consistent with reactive colonic glands. The patient remained asymptomatic after one-year follow-up. A review of the literature shows very rare descriptions of similar lesions, but none fits exactly this pattern. We would designate this inverted lymphoglandular polyp and present this case to raise the awareness of recognizing this unusual histological entity.

## 1. Introduction

When glandular epithelium appears in the submucosa or the deeper layers of the intestinal wall, it raises the potential diagnostic confusion with other more serious lesions, such as adenomas with pseudoinvasion or well-differentiated adenocarcinoma. Here, we present a descending colonic sessile polyp, which shows an inverted pattern with an intimate admixture of cystically dilated columnar epithelium lined glands and lymphoid aggregates with germinal centers in the submucosal layer. To date, there have been no reports of the same type of colonic polyp as that reported here.

## 2. Case Presentation

A 47-year-old male with a history of left colon cancer, status post left colon resection for 12 years, presented for surveillance examination and recent rectal bleeding. He had no other symptoms or family history. He underwent esophagogastroduodenoscopy and colonoscopy. The esophagogastroduodenoscopy showed normal vocal cords, esophagus, GE junction, stomach, duodenal bulb, and 2nd portion of the duodenum. On colonoscopy, there was a 3 mm sessile polyp in the cecum, which was ablated with snare tip, an 8 mm sessile polyp in the proximal descending colon, which was snared with electrocautery and retrieved, and a 7 mm sessile polyp in the proximal rectum, which was also snared and retrieved. The patient had an uneventful postoperative course. The patient remained asymptomatic after one-year follow-up.

**Figure 1:**
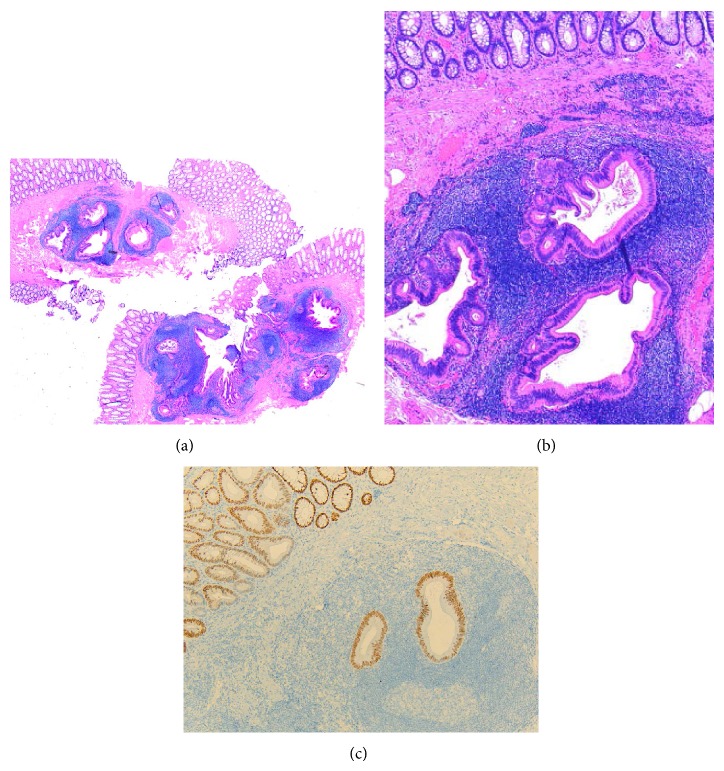
Pathologic findings. (a) At low power, an inverted growth of the cystically dilated glands with prominent lymphoid aggregates, similar to a greatly expanded lymphoglandular complex in the submucosa. (b) At higher power, the glands were lined by columnar epithelium with basally located small nuclei and reactive changes and surrounded by lymphoid aggregates. Note the absence of serrated tubular or adenomatous features or goblet cells. (c) Immunohistochemical staining showed the epithelial element was positive for CDX2 (CDX2 immunostaining).

The descending colon polyp was received in formalin and measured 0.6 × 0.2 × 0.2 cm. The specimen was bisected and totally embedded in one cassette. Paraffin embedded tissue was sectioned at 5 microns and multiple levels were evaluated with routine hematoxylin and eosin stain. Microscopic examination showed that most of the surface of this polyp was covered with a layer of normal mucosa with muscularis mucosae, and focal erosion was found on one part of the surface. In the submucosal layer, an intimate admixture of multiple cystically dilated glands and prominent lymphoid aggregates with germinal centers was seen. The glands were lined by columnar epithelium with basally located small nuclei. No goblet cells were present. The lymphoid aggregates surrounded the individual glands (Figures 1(a) and 1(b)). No acute inflammation or hyperplastic or adenomatous changes were seen. The deep and lateral aspects of the polyp were well circumscribed without any invasive tendency. Immunohistochemical staining showed the epithelial element was positive for CK20 and CDX2 (Figure 1(c)) and negative for CK7, with a low proliferative index (less than 1% of cells staining of Ki67), mostly consistent with reactive colonic glands. The rectum polyp is an adenomatous polyp.

## 3. Discussion

A review of the literature shows very rare descriptions of similar lesions, but none fits exactly this pattern. We would designate this inverted lymphoglandular polyp.

Our first impression of the lesion is colitis cystica profunda. Colitis cystica profunda is a rare benign condition characterized by cystic dilation and misplacement of mature crypts through the muscularis mucosa and/or deeper layers of the bowel wall. Colitis cystica profunda is generally localized and only rarely diffuse [1]. The localized form is usually localized to the rectum and presents as nodule or plaque, associated with the solitary rectal ulcer syndrome [2, 3]. Glands entrapped in the walls of the intestine commonly undergo dilatation and cystic change and often have a loss of epithelium because of pressure atrophy. And the diffuse type is often secondary to inflammatory bowel disease. It is characterized by mucus containing cyst in colonic submucosa caused by mucosa prolapse as a result of mucosal inflammation and regeneration. The location and absence of significant inflammation change of our case argue against this entity.

The submucosal growth pattern of inverted lymphoglandular polyp is similar to a case report of “inverted colonic mucosal lesion” [4]. However, in that case, the gross configuration of the lesion is a depressed one, and inverted glands in the submucosa were similar to the normal colonic mucosal glands. In our case, the inverted glands are cystically dilated, lined by columnar epithelium without goblet cells, located in deep submucosa, and morphologically different from the overlying normal mucosal glands.

An intimate admixture of lymphoid aggregates and columnar epithelial glands of inverted lymphoglandular polyp resembles lymphoglandular complex [5, 6]. Lymphoglandular complex is a normal structural entity of the large bowel and it acts as a local receptor of antigenic material for future immune recognition. Lymphoglandular complexes are in close apposition to the muscularis mucosae, either above it in the lamina propria or below it in the upper part of the submucosa. The glandular components of lymphoglandular complex are intestinal glands which were indistinguishable from corresponding cells lining mucosal glands nearby. Although lymphoglandular complexes are plentiful in the ascending colon, they are relatively fewer in number in descending colon.

Inverted lymphoglandular polyp is also different from inverted hyperplastic polyp. The latter is characterized by proliferative tubules in submucosa with surface mucus hypersecretion [7].

Tanaka et al. [8] reported a polypoid colonic hamartomatous inverted polyp. Hamartomatous inverted polyps are benign masses formed by an inverted or downward growth of mucosal glands through the muscularis mucosa into the submucosa, occurring in the rectum. Microscopically, ectopic distorted, slightly atypical mucous glands are present in a more or less circumscribed group in the submucosa, pushing the mucosa up to form a polypoid mass. Hamartomatous inverted polyps may simulate this microscopically but are different from this present case in terms of their gross configuration and location and without lymphoid aggregates.

The mechanism of inverted lymphoglandular polyp is unclear. We propose that this kind of polyp may result from local trauma and subsequent protrusion of mucosal gland into the submucosa through inherently weak areas in the muscularis mucosae, such as that which occurs adjacent to lymphoid aggregates.

Lack of cellular atypia and low proliferative index suggests that the neoplastic potential of inverted lymphoglandular polyp is likely to be very low. However, since this is such a rare case, it is uncertain whether it needs to be removed or simply just followed up.
